# Tuning Thermal and Mechanical Properties of Polydimethylsiloxane with Carbon Fibers

**DOI:** 10.3390/polym13071141

**Published:** 2021-04-02

**Authors:** Nevin Stephen Gupta, Kwan-Soo Lee, Andrea Labouriau

**Affiliations:** C-CDE Chemical Diagnostics and Engineering, Los Alamos National Laboratory, Los Alamos, NM 87545, USA; nevin5@lanl.gov

**Keywords:** Sylgard™ 186, polydimethylsiloxane, carbon nanofibers, hardness, young’s modulus, degradation temperature, coefficient of thermal expansion

## Abstract

In order to meet the needs of constantly advancing technologies, fabricating materials with improved properties and predictable behavior has become vital. To that end, we have prepared polydimethylsiloxane (PDMS) polymer samples filled with carbon nanofibers (CFs) at 0, 0.5, 1.0, 2.0, and 4.0 CF loadings (*w*/*w*) to investigate and optimize the amount of filler needed for fabrication with improved mechanical properties. Samples were prepared using easy, cost-efficient mechanical mixing to combine the PDMS and CF filler and were then characterized by chemical (FTIR), mechanical (hardness and tension), and physical (swelling, thermogravimetric analysis (TGA), differential scanning calorimetry (DSC), and coefficient of thermal expansion) analyses to determine the material properties. We found that hardness and thermal stability increased predictably, while the ultimate strength and toughness both decreased. Repeated tension caused the CF-filled PDMS samples to lose significant toughness with increasing CF loadings. The hardness and thermal degradation temperature with 4 wt.% CF loading in PDMS increased more than 40% and 25 °C, respectively, compared with the pristine PDMS sample. Additionally, dilatometer measurements showed a 20% decrease in the coefficient of thermal expansion (CTE) with a small amount of CF filler in PDMS. In this study, we were able to show the mechanical and thermal properties of PDMS can be tuned with good confidence using CFs.

## 1. Introduction

Polydimethylsiloxanes (PDMSs), also called siloxanes or silicones, are widely used, silicon-based organic polymers that have backbone flexibility, low intermolecular interactions, low surface tension, biocompatibility, and thermal stability [1,2,3]. These properties make them appealing for many applications, such as textiles, coatings, construction, adhesive, electronics, photonics, transportation, and medical applications [4,5,6,7,8,9,10,11,12,13,14]. However, PDMS has certain mechanical properties that limit its use in several applications. Two of the greatest challenges are the low elastic modulus and high coefficient of thermal expansion (CTE) [15]. As an example, PDMS’s high CTE limits its applicability in electronic circuits, which have become faster and denser, requiring materials that are capable of dissipating heat and retaining their origin shape. Unmodified PDMS is not able to keep up with these improvements in circuit technology [14,16,17]. Therefore, tuning PDMS’s properties and processability with optimized filler loadings is necessary, in particular, where coating, sealing, potting, and casting fabrication processes are required.

In order to overcome the mechanical and thermal shortcomings of PDMS, extensive research has focused on improving the material properties using different fillers. Carbon nanofibers and nanotubes [18,19,20], graphite [21], graphene [22], nanodiamond [23], carbon black [18], silica [24], nanoclay [25], and other fillers have all been used to improve the properties of PDMS and other polymer materials. Specifically, carbon fibers have several qualities that make them an appropriate filler for polymer materials. They have excellent tensile properties (high strength and high modulus), thermal and electrical conductivities, and low density [26,27,28]. The tensile strength and Young’s Modulus from carbonized polyacrylonitrile were reported as 300–600 MPa and 40–60 GPa, respectively [29]. Furthermore, previous investigation has shown that a 10 wt.% carbon nanofiber loading can decrease the CTE of an epoxy resin by 11.6% compared with neat epoxy [30]. Based on the improved properties reported in previous studies, carbon fibers were chosen for this study to be added to PDMS-based materials due to their excellent mechanical and thermal properties (mechanical strength, Young’s Modulus, and CTE).

Here, we present the mechanical, thermal, and dimensional stability behavior of Sylgard™ 186, a PDMS polymer, filled with varying CF loadings. Prior to fabricating the materials for mechanical and thermal characterization, rheological analysis of the CF-filled PDMS composites was employed to evaluate whether the viscoelastic properties were appropriate to fabricate devices for various engineering applications. In addition, rheology was used to confirm the dispersion of CFs in the PDMS matrix. Five different CF concentrations were used to understand the behavior of CF-filled PDMS in this study: PDMS pristine (i.e., Sylgard™ 186), 0.5, 1.0, 2.0, and 4.0 wt.% CF in PDMS (*w*/*w*). During our review of the literature, we found that the vast majority of studies used the low molecular weight Sylgard™ 184 PDMS polymer (Component A: M_n_ = 5.8 × 10^3^ Da; Component B: M_n_ = 7.5 × 10^3^ Da). Few studies looked at the behavior of the higher molecular weight Sylgard™ 186 polymer (Component A: M_n_ = 4.8 × 10^6^ Da; Component B: M_n_ = 1.6 × 10^6^ Da) [31]. Therefore, Sylgard™ 186 was chosen as the polymer matrix to provide a good guideline for tuning relevant properties with CF fillers.

## 2. Materials and Methods

### 2.1. Materials

**Scheme 1:**
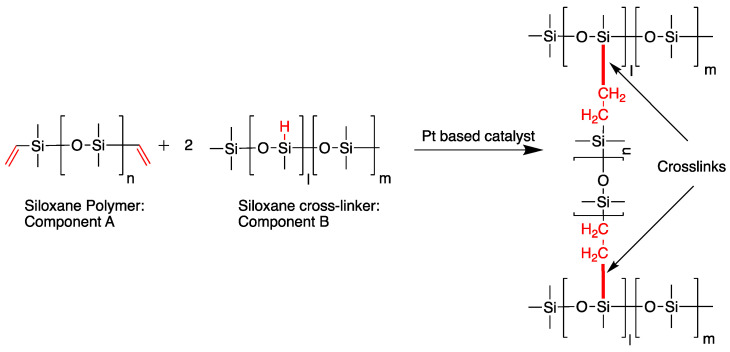
Hydrosilylation reaction between vinyl-terminated polydimethylsiloxane and methylhydrosiloxane-dimethylsiloxane copolymer [32,33]. Crosslinking occurs between the vinyl terminated group from Component A and the Si–H group from Component B in the presence of the Pt based catalyst. The chemical bonds active in crosslinking are shown in red, and the thick red line indicates the newly formed crosslinks.

Polydimethylsiloxane (Dow Sylgard™ 186) was supplied by Ellsworth Adhesives (Germantown, WI, USA). The base (or component A) to curing agent (or component B) ratio used was 10:1, *w*/*w*, as specified by Dow, and was kept constant for all formulations. Sylgard™ 186 is an addition cure-type PDMS elastomer comprising two components. Curing, which is a direct result of crosslinking, occurs when component A (elastomer base), a viscous liquid containing vinyl-terminated polydimethylsiloxane, is combined with component B (elastomer curing agent), a less viscous liquid containing a platinum catalyst and a methylhydrosiloxane-dimethylsiloxane copolymer (Scheme 1) [32,33].

Carbon nanofibers (PR-19-XT-PS) were supplied by Pyrograf Products, Inc. (Cedarville, OH, USA). The fibers have a chemically vapor-deposited layer of carbon over a graphitic tubular core fiber and have an average diameter of 150 nm and an estimated length between 50 and 200 µm [34]. HPLC-grade toluene (Thermo Fisher Scientific, Waltham, MA, USA) was used for solvent swelling experiments. All materials and chemicals were used as received.

### 2.2. Fabrication of Carbon Nanofiber/Polydimethylsiloxane (CF/PDMS) Composites

Samples were first prepared by, first, hand mixing the Sylgard™ 186 base with the appropriate CF content until the dispersion had become visually homogenous. After mixing, the curing agent was added and then hand mixed until visually homogenous (~30 s). The mixture was then immediately mixed using an ARV-310 Thinky Planetary Mixer (THINKY USA Inc., Laguna Hills, CA, USA)—samples were placed in a chilled container during mixing to reduce heating and subsequent premature curing during mixing—at 2000 rpm under a vacuum for 2 min (Thinky container, THINKY USA Inc., Laguna Hills, CA, USA). Hardness samples (buttons) were cured at room temperature (25 ± 3 °C) in Teflon molds (29 mm diameter, 13 mm deep). The hardness change of the samples was observed with time to determine the room temperature curing characteristics. Sheets for tension, swelling, thermogravimetric analysis (TGA), and differential scanning calorimetry (DSC) were cured in aluminum molds (4.25 in length, 3 in width, 1 mm deep) lined with polytetrafluoroethylene (PTFE) glass cloth tape (3M, 5151–5153) at 150 °C for 30 min. For the CTE experiment, the mixed samples were placed in a syringe and centrifuged (Thermo Fisher Scientific, Megafuge 8) at 1000 rpm for 1 min 30 s to remove air bubbles. Samples were then pipetted into a rod-shaped Teflon mold (size: 5.0 mm in diameter and 20.0 mm in length) and cured at 150 °C for 30 min. In this work, Sylgard™ 186, and 0.5, 1, 2, and 4 wt.% of CF filler in Sylgard™ 186, are referred to as PDMS pristine, CF-0.5/PDMS, CF-1.0/PDMS, CF-2.0/PDMS, and CF-4.0/PDMS, respectively, and carbon-filled PDMS composites are referred to as CF/PDMSs.

### 2.3. Characterization and Measurements

#### 2.3.1. Tension

Tension (or tensile) tests were carried out to understand how the CF/PDMS composites would behave initially and after being pulled to 100% elongation. Samples were cut using a Type-A die (ASTM D412) and run on the ADMET eXpert 7601 (Norwood, MA, USA) testing system. The samples were placed in ADMET vice grips with a gap of 50 mm. The samples were loaded so they were barely taught (no visual sag nor noticeable tension). The samples were then pulled to 100 mm at 3.84 mm/s and returned to the 50 mm home immediately. The samples were then pulled until failure at 3.84 mm/s. The toughness was calculated using the integral of the area under the stress-strain curve. Samples were 1.00 ± 0.15 mm in thickness.

#### 2.3.2. Hardness

Hardness tests were run according to the methods noted by Murphy and coworkers [6]. Samples were kept in a dark environment at 25 ± 3 °C for the duration of the experiments. Three measurements were performed on three different locations of a given button, and three different buttons for each of the pristine and CF/PDMS composites were tested. The value and error reported are the average and standard deviation, respectively.

#### 2.3.3. Thermogravimetric Analysis (TGA)

Samples of 5–10 mg were run on a TA Instruments Discovery Series 550 (New Castle, DE, USA). The samples were first equilibrated at 50 °C, then heated to 900 °C at a heating rate of 10 °C/min under a nitrogen atmosphere. The degradation temperature is reported as the temperature at which 5% mass loss occurred. Peak degradation temperatures were found using the first derivative graphs.

#### 2.3.4. Differential Scanning Calorimetry (DSC)

Measurements were taken using a TA Instruments Discovery Series 2500, with a 5–10 mg sample. The following procedure was used: heating from −150 °C to 100 °C at a heating rate of 10 °C/min (1st heating ramp), 5-min isothermal, cooling from 100 °C to −150 °C at a cooling rate of 10 °C/min, 5-min isothermal, and heating −150 °C to 100 °C at a heating rate of 10 °C/min (2nd heating ramp). The second heating ramp was used for analysis. The glass transition temperature (T_g_), melting temperature (T_m_), crystalline temperature (T_c_), enthalpy of melting (ΔH_f_) and crystallization enthalpy of crystallization (ΔH_c_) values were calculated using TRIOS software. Crystallinity was calculated using the equation below (61.19 J/g is the theoretical enthalpy of fusion for a fully crystalline PDMS polymer) [35]:

(1) $$\chi \;\left(\%\right)=\;\frac{\Delta H_{f}}{61.19}\times 100$$

#### 2.3.5. Solvent Swelling Ratio (%)

Discs (12.7 mm diameter, 1.00 ± 0.15 mm thick) were swollen in excess toluene in a sealed vial at room temperature for one week in a dark environment. Samples were then dried at 70 °C under a vacuum for 24 h. The swelling ratio was calculated using the equation below:

(2) $$Swelling\;ratio\;\left(\%\right)=\frac{m_{w}-m_{d}}{m_{d}}\times 100$$

where *m_w_* is the swollen mass, and *m_d_* is the dried mass. Samples were run in triplicate and standard deviation is reported as the error.

#### 2.3.6. Fourier-Transform Infrared (FTIR) Spectroscopy

A Nicolet iS50 FT-IR from Thermo Fisher Scientific was used for FTIR analysis. 32 scans were taken, giving a resolution of 4 cm^−1^. A range of 4000 cm^−1^ to 500 cm^−1^ was used. The spectra were normalized using the Si–CH_3_ peak at 780 cm^−1^.

#### 2.3.7. Rheology

Rheological measurements were conducted using a Discovery Hybrid Rheometer-2, a TA Instruments rheometer using parallel plate fixtures with a diameter of 25 mm. To evaluate the viscoelastic properties of PDMS pristine and CF/PDMS composites, stress sweeps were performed in the range of 10 to 6500 Pa at a fixed angular frequency of 10 rad/s for each measurement.

#### 2.3.8. Coefficient of Thermal Expansion (CTE)

The CTE rods were tested using a TA Instruments DIL 802 dilatometer. Samples were heated from room temperature to 150 °C at 5 °C/min under a nitrogen atmosphere. Sapphire rods were used as a standard to calibrate the instrument. All samples showed a relatively constant alpha (CTE) value between 60 °C and 75 °C; therefore, this region was used to calculate the alpha for all samples. Samples were 5.0 ± 0.5 mm in diameter and 20.0 ± 1.0 mm in length.

## 3. Results and Discussion

### 3.1. Preparation of Carbon Fiber Filled Sylgard™ 186

Understanding the rheological behavior (e.g., viscoelastic properties, viscosity) of carbon fiber filled PDMS (CF/PDMS) composites is essential to prepare samples and fabricate devices for various engineering applications, particularly sealing, casting, and potting applications. PDMS pristine and CF/PDMS composites were prepared to investigate rheological behavior; namely, how increasing the CF content affects the mechanical properties of the final product.

**Figure 1:**
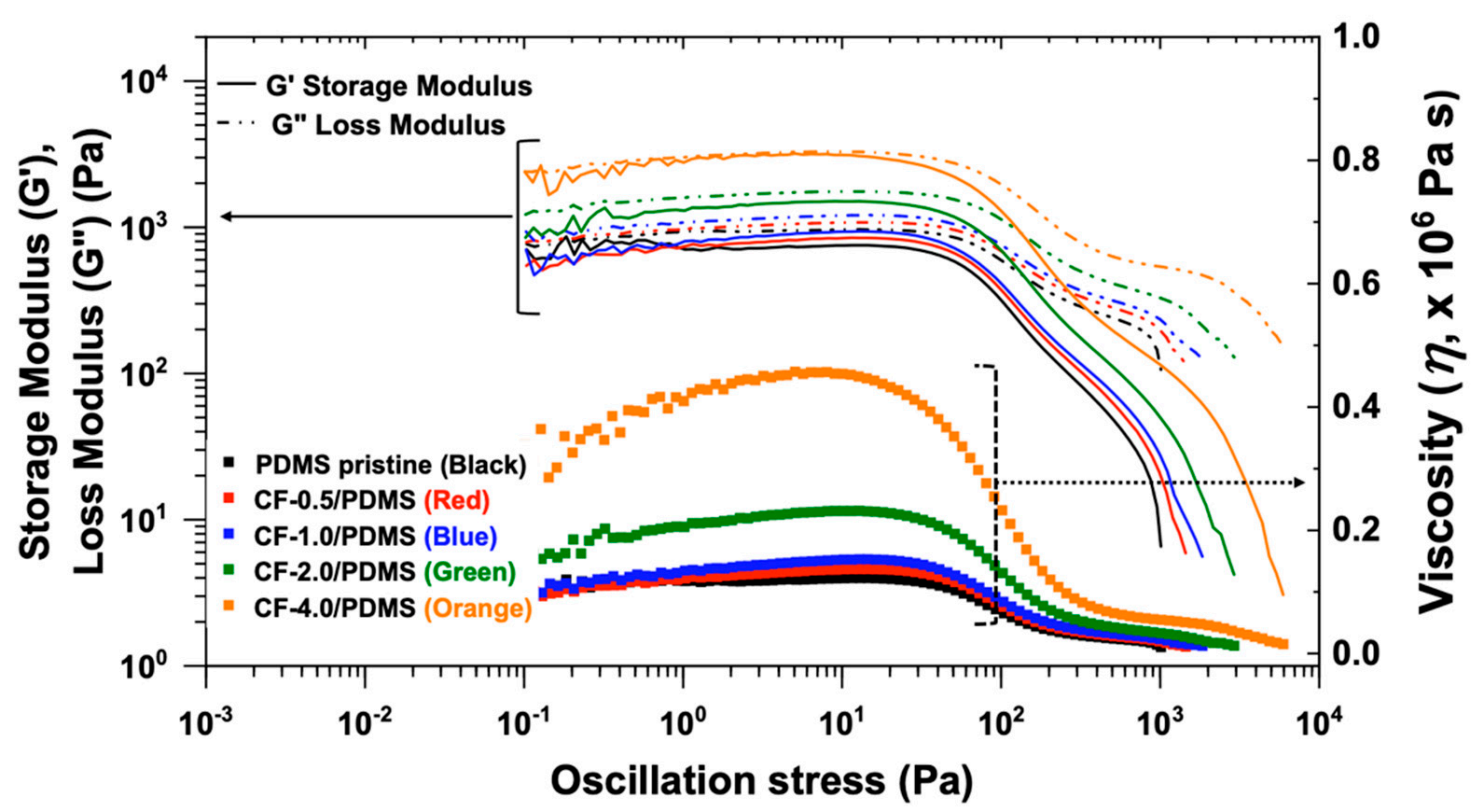
Rheological properties of polydimethylsiloxane (PDMS) pristine and CF/PDMS composites as a function of carbon nanofibers (CF) contents (0.5, 1.0, 2.0, and 4.0 wt.%).

We selected 0.5, 1, 2, and 4 wt.% CF filler in PDMS to compare with PDMS pristine as a control sample. As shown in Figure 1, the solid-like property (G’: storage modulus) of CF-4.0/PDMS is very close to the liquid-like property (G”: loss modulus) (G’ ≈ G”), even with a significant increase in the viscosity. This demonstrates that 4 wt.% CF filler in PDMS is too thick, indicating that it is not appropriate for material fabrication processes requiring low viscosity materials. In addition, shear thickening behavior, where G’ increases with increasing oscillation stress, has not been observed, which indicates the CF fillers are well dispersed in the PDMS matrix without filler clustering/rearrangement [36].

### 3.2. FTIR Spectroscopy

**Figure 2:**
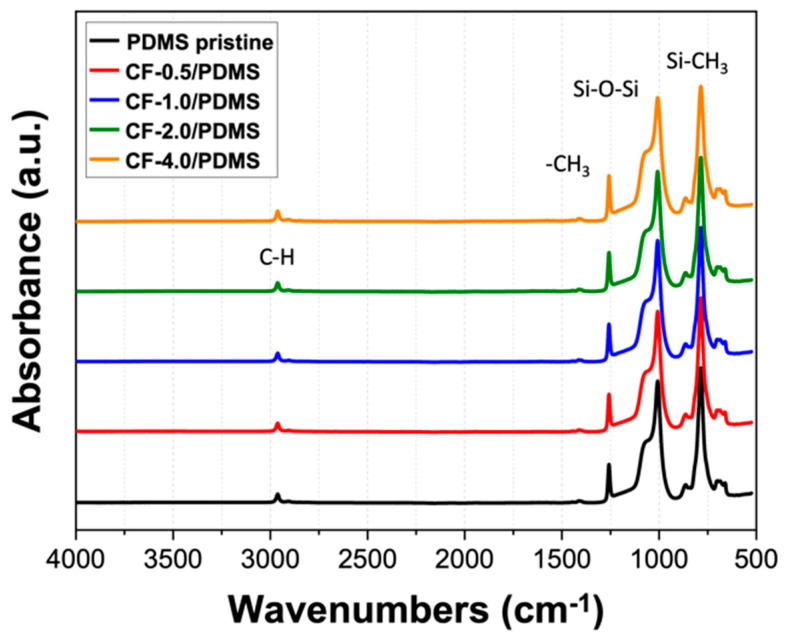
FTIR spectrum of crosslinked CF filled PDMS, normalized with the Si–CH_3_ peak.

The FTIR for all samples showed excellent agreement with previous FTIR results for PDMS [12,37,38]. Several characteristic peaks were identified and are shown in Figure 2: the peak at 2965 cm^−1^ corresponds to the C–H stretch, the peak at 1260 cm^−1^ corresponds to the –CH_3_ deformation in Si–CH_3_, the peaks at 1400 and 1420 cm^−1^ correspond to –C–H bending, the peak between 930 and 1200 cm^−1^ corresponds to Si–O–Si stretching, the peak between 825 and 865 cm^−1^ corresponds to the Si-C rocking peak, and the peak between 785 and 815 cm^−1^ corresponds to the Si–CH_3_ rocking peaks. In addition, a peak at around 2200 cm^−1^, corresponding to Si–H stretching, was not observed for any sample, as shown in Figure 2, verifying that the crosslinking reaction (i.e., hydrosilylation reaction shown in Scheme 1) reacted and a similar high crosslink density was achieved for all samples [39].

### 3.3. Effects of the CF Filler on Mechanical Properties and Dimensional Stability

**Figure 3:**
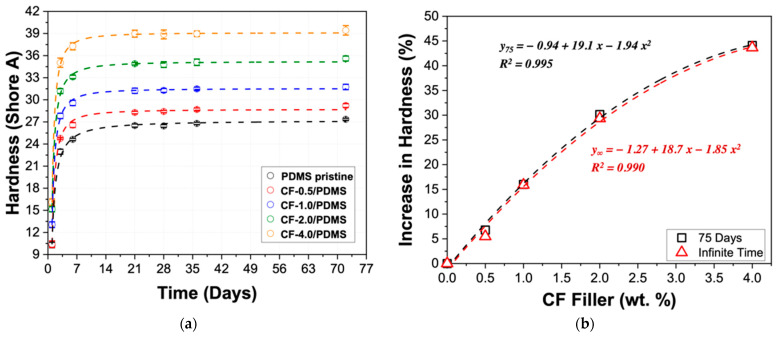
(**a**) Shore A hardness for CF-filled PDMS samples from 1 to 72 days; data fit using logistic curves. (**b**) Percent increase in hardness compared with PDMS pristine. The black data show an increase in hardness using 72-day hardness, and the red data show an increase in hardness at infinite time, determined using fits from the left graph.

Hardness tests were used to understand how the material behaves over time, or more specifically, how the final hardness changes and how the CF filler affects the curing of PDMS. It is important to note that these samples were cured at room temperature; therefore, the crosslinking reaction was ongoing for these samples. Figure 3a shows the change in hardness over 72 days. All of the samples exhibit a sharp increase in hardness over the first three to four days. After this sharp increase, the samples continue to increase but much slower, almost reaching a steady state after 21 days. There is no discernable difference in the shape of the curing curves, which suggests the CF filler does not disrupt the curing of the polymer matrix itself. Although an apparent steady state has been reached for all the samples after about 21 days, the PDMS pristine and the CF-0.5/PDMS samples showed a statistically significant increase in hardness, even after 72 days. It was observed that these samples were still curing at 72 days.

Each data set was fit using a logistic curve and shows good agreement with all CF loadings—all R^2^ values are greater than 0.985. Using the fits shown in Figure 3a, the hardness at infinite time were calculated for all samples: 27.2 ± 0.5, 28.7 ± 0.4, 31.5 ± 0.3, 35.2 ± 0.4, and 39.1 ± 0.2 were determined for PDMS pristine, CF-0.5/PDMS, CF-1.0/PDMS, CF-2.0/PDMS, and CF-4.0/PDMS, respectively. Dow reports the Shore A hardness of Sylgard™ 186 as 24 [40]. This difference can be explained by the curing conditions (humidity, temperature, etc.), which have been shown to have a significant effect on the final mechanical, thermal, and physical properties of PDMS [41,42,43]. Figure 3b shows the percentage increase in hardness when compared with PDMS pristine after 72 days (black squares) and at their ultimate hardness (hardness at infinite time, values given above; red triangles). The agreement between the black and red fits means that, despite continued crosslinking by the PDMS pristine and the CF-0.5/PDMS sample, the materials’ hardness can be confidently tuned within the given CF loadings for its extended use in various applications by applying the simple second degree polynomial equations shown in Figure 3b.

In order to understand how the elastic properties of PDMS change with the addition of CF fillers, tension tests were performed. Table 1 shows the resulting elongation, ultimate strength, toughness, and Young’s Moduli with increasing CF loadings. Previous investigations using CFs as a filler material in other polymer matrices have found that the alignment of CFs in the polymer matrix plays a significant role in how the mechanical properties of the material change [44,45,46]. Specifically, they note that the greatest increases in mechanical properties are observed when the CF fillers are aligned parallel to the applied force. As we discussed previously in relation to the rheological properties of CF/PDMS composite formulations, CF fillers in the PDMS are well dispersed, indicating that their orientation in the PDMS matrix was random. The results of the tension tests for the CF/PDMS samples showed a decrease in the elongation, ultimate strength, and toughness, but an increase in the Young’s Modulus with increasing CF loadings. Microscopic investigation in previous works showed that holes, voids, and other defects are formed by moving CFs in the PDMS polymer matrix when stress was applied to the material [44,47]. The accumulation of these defects in the CF-containing samples likely caused the sample to break before reaching the same ultimate strength and elongation as the PDMS pristine. This decrease in ultimate stress and elongation led to a decrease in toughness.

**Figure 4:**
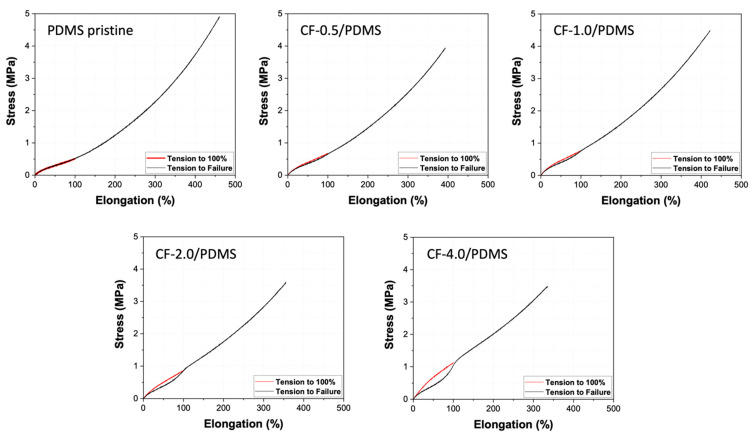
Stress-strain curves as a function of CF filler amount in PDMS matrix. Red curves show initial tension from 0% to 100%, and black curves show subsequent tension on the samples previously elongated to 100% until failure.

**Figure 5:**
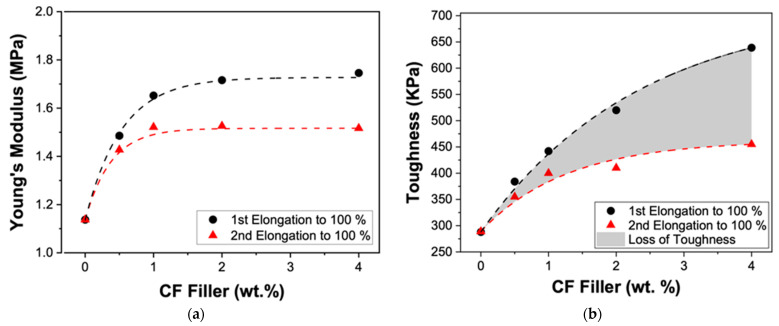
(**a**) Young’s Modulus and (**b**) toughness change as a function of the amount of CF filler. Young’s Modulus and toughness values are from the stress-strain curves of samples for the 1st trials (black circles; 1st elongation to 100%) and the 2nd trials (red triangle; 2nd elongation to 100%). The gray area is the loss of toughness.

This study also explored how the CF filler affects repeated tension performance (Table 1 and Figure 4). The samples were first elongated to 100% (red curves in Figure 4) and then elongated a second time until they failed (black curves in Figure 4). Since the region between 0% and 100% elongation is within the elastic domain of PDMS pristine, there is no difference between the first and second trials for the PDMS pristine, as expected. It is clear that as the amount of CF increases, the separation between the red and the black curves increases, denoting that the polymer is no longer fully elastic in this region, which we attribute to the defects caused by the CF filler, as mentioned before. In Figure 5, to evaluate the mechanical behavior changes in the range of 0% to 100%, the differences in Young’s Modulus and toughness are displayed. Interestingly, both the initial trial and the second trial (tension test after 100% initial elongation) showed a threshold point at 2 wt.% CF filler. At this stage, the Young’s Moduli (initial and second trial) reached an apparent steady state, and additional CF showed no clear increase in the Young’s Moduli, signifying 2 wt.% CF loading in this PDMS (i.e., Sylgard™ 186) is the optimum concentration for a high Young’s Modulus value, even after repeated tension testing.

There is a significant loss of toughness (in the range of 0% and 100%) seen in all of the CF/PDMS samples between the first and second elongation, as shown in Figure 5b. This loss in toughness between the 1st and 2nd trials also supports the reasoning that the 2 wt.% loading of CF filler is the optimal loading for improved mechanical properties: 2 wt.% is the point where the Young’s Modulus appears to level off, while minimizing the loss of toughness. While the CF-4.0/PDMS sample has a slightly larger initial Young’s Modulus, the loss of toughness is much greater than the CF-2.0/PDMS. This may limit the applicability of the CF-4.0/PDMS sample.

**Figure 6:**
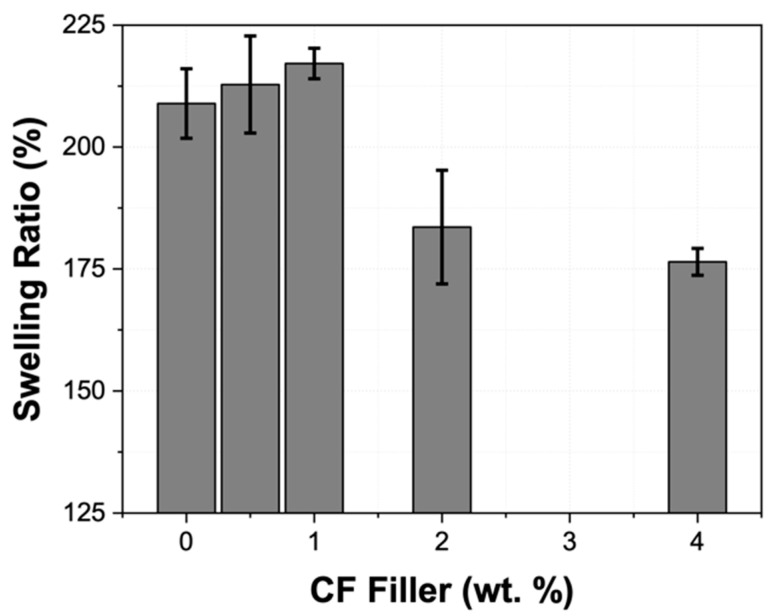
Swelling ratio (%) as a function of the amount of CF filler in the PDMS matrix.

Solvent swelling tests were used to probe the effect of the CF filler on the PDMS crosslink density. As shown in Figure 6, the PDMS pristine, CF-0.5/PDMS, and CF-1.0/PDMS samples show statistically similar swelling after 1 week, while the CF-2.0/PDMS and CF-4.0/PDMS samples both show significantly decreased swelling compared with PDMS pristine. In general, a decrease in the swelling ratio is associated with an increase in crosslink density; however, the FTIR analysis mentioned previously showed no Si–H peak for any samples, indicating that all samples had reached a similar high degree of crosslinking. Consequently, crosslinking density would not explain the results as shown in Figure 6. This result suggests that the CF filler (≥2 wt.%) acts as a physical cross-linker in the PDMS matrix, therefore increasing the dimensional stability of the samples. In addition, since there is no change in the chemical bonding and crystallinity (FTIR and DSC), nor in the crosslinking (hardness), the CF filler must only have weak interactions with the PDMS polymer matrix [48]. Thus, we infer that the CF filler only fills the free volume between the polymer molecules in the matrix, partially inhibiting the solvent from completely entering the matrix during swelling. Swelling at equilibrium is reached when the chemical potential gradient forcing the solvent into the PDMS polymer matrix (diluting the monomers in the polymer, thus achieving greater entropy) is balanced by the elasticity of the polymer network, which resists infinite deformation [49]. In the CF-0.5/PDMS and CF-1.0/PDMS samples, there was a slightly greater chemical potential forcing the solvent into the polymer network than the PDMS pristine sample due to the increase in entropy from diluting the CFs. However, this force was balanced by the slight extension in the polymer network caused by the CF filler, which we attribute as the reason there is no change between the PDMS pristine, CF-0.5/PDMS, and CF-1.0/PDMS samples. In the CF-2.0/PDMS and CF-4.0/PDMS samples, however, the distension of the polymer network resisting swelling dominates the increased entropy associated with diluting the CFs; therefore, there is a significant decrease in the swelling ratio.

These data show that there is a CF loading limit between 1.0 and 2.0 wt.% where the CFs inhibit the solvent from entering and swelling the PDMS network, indicating good chemical resistance and dimensional stability with ≥2 wt.% loading of CF filler in PDMS.

### 3.4. Effect of the Amount of CF Filler on Thermal Behaviors

**Figure 7:**
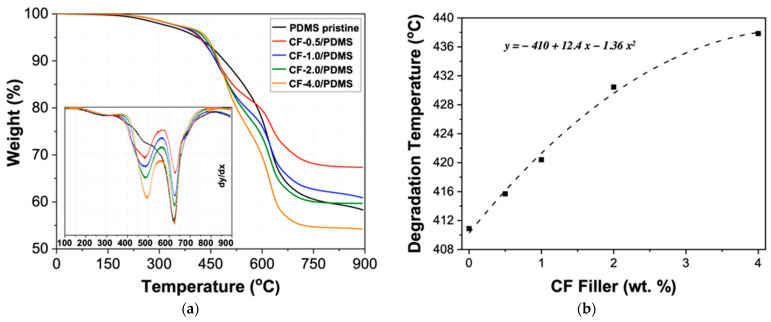
(**a**) Thermogravimetric analysis (TGA) for PDMS pristine and CF/PDMS samples. Inset: derivative curves. (**b**) Degradation temperature (Td; 5% weight loss).

The thermal degradation behavior of the PDMS pristine and CF/PDMS samples was monitored using thermogravimetric analysis (TGA), as shown in Figure 7 and Table 2. The degradation exhibits three steps for all samples, which can be seen from the three peaks in the derivative graph in Figure 7a. Zhou and coworkers showed a similar degradation profile for PDMS. The first degradation is a slow degradation step at about 300 °C [50]. This degradation occurred before the degradation temperature (T_d_), denoted by a 5% weight loss, was reached, and we attribute this to the loss of small molecules or non-crosslinked polymer chains trapped in the polymer matrix. After this step, the samples reach their degradation temperatures. In Figure 7b, there is an increase in the degradation temperature with increasing CF content. Similar to the increase in hardness (Figure 3b), the increase in degradation temperature follows a second-degree polynomial equation, shown in Figure 7b, with high agreement (high R^2^ value = 0.991). Using this information, the thermal degradation of CF/PDMS can be tuned to meet the needs of specific applications.

Additionally, there are two more degradation peaks seen in the derivative graph, which occur at ~480 and ~625 °C. The peak temperature for both of these degradation steps does not appear to be significantly altered by the presence of the CF filler. However, the first degradation step, which is present in the PDMS pristine curve, becomes increasingly pronounced with the addition of CF fillers. Based on the increase in degradation with increasing CF filler, we ascribe this degradation temperature to the CF filler. In a previous study, styrene filled with CFs showed the CF degradation temperature to be between 480 °C and 550 °C, which supports our findings [51]. The third degradation temperature is the maximum degradation peak for all samples, which we attribute to depolymerization of PDMS [52].

**Figure 8:**
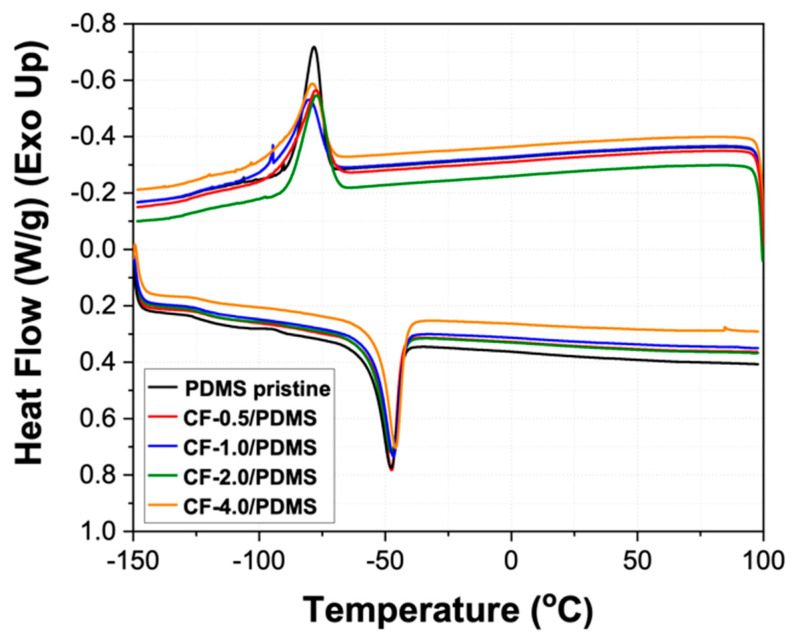
Differential scanning calorimetry curves for PDMS pristine and CF/PDMS samples.

Differential scanning calorimetry (DSC) measurements were run to evaluate how the incorporation of the CF filler affected the glass transition temperature (T_g_), melting temperature (T_m_), enthalpy of fusion (ΔH_f_), crystallization temperature (T_c_), and enthalpy of crystallization (ΔH_c_). Figure 8 shows the DSC curves for all the samples, and the resulting values are given in Table 3. As shown in Table 3, there is no significant change in any of the given values. As shown in Figure 8, there is a single endothermic peak in both the cooling ramp (top curves) and the heating ramp (bottom curves), corresponding to a single crystalline structure. There is no broadening or shift in either the fusion peak or the crystallization peak, which indicates a similar crystalline structure in all samples. Since formation of the crystalline structure is largely dependent on the steric stresses, it is clear the CFs do not have any significant bonding/interaction with the polymer matrix, as these interactions would have increased the steric hindrance of the polymer molecules [53]. Furthermore, as Table 3 shows, there is also no significant change in the degree of crystallinity (38%).

This indicates that the CF filler has no effect on the formation of the polymer matrix, and there are only weak interactions (i.e., Van der Waals) between carbon nanofibers and the PDMS polymer. Furthermore, the minute changes in the glass transition temperature without exothermic peaks in the DSC curves indicate an equal and high degree of crosslinking in all the samples [48].

**Figure 9:**
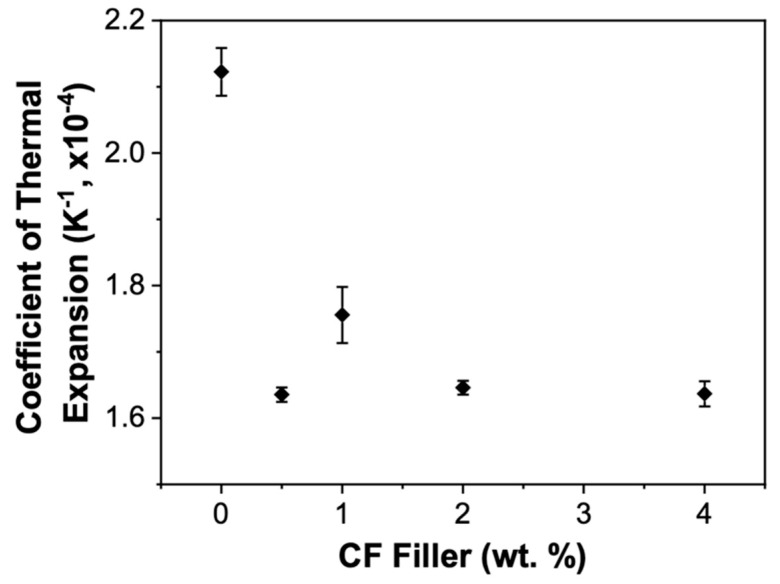
Coefficient of thermal expansion as a function of the amount of CF filler in the PDMS matrix.

Lastly, the coefficient of thermal expansion (CTE) was determined. Figure 9 shows the CTE values for the different CF loadings. The CTE given by Dow is 3.3 ×10^−4^ K^−1^ for Sylgard^TM^ 186 [40]. In this study, however, a value of 2.12 ± 0.06 × 10^−4^ K^−1^ was attained. As mentioned before, the hardness of the PDMS pristine sample attained in this study was slightly higher than the reported value by Dow. Differences in the curing conditions have been shown to affect the final properties of PDMS [41,42,43], and it is consistent to have a lower CTE value, given that the PDMS showed a slightly higher hardness value.

The more significant result, however, is how the CF filler affects the CTE. These data show a significant decrease (20%) in the CTE for the CF-0.5/PDMS sample. Thus, a significant reduction in CTE can be achieved with minimal CF filler, making CF-filled PDMS more applicable to certain applications such as potting material for electronics. The other three samples show similar CTE values as the CF-0.5/PDMS sample and exhibit little dependence on the CF concentration.

## 4. Conclusions

In summary, carbon nanofiber (CF)-filled PDMS samples were fabricated to evaluate how CFs would affect the mechanical behavior, thermal properties, and dimensional stability of PDMS. Rheological behavior of CF/PDMS composite formulations showed that the CF fillers were well dispersed in the PDMS polymer matrix without rearrangement of the filler. Hardness values were increased with increasing CF loadings in PDMS. In particular, they were significantly increased by more than 40% (39.1) with the addition of 4 wt.% CF filler in PDMS, compared with that of the PDMS pristine (27.2). The results of the tension tests displayed a decrease in the elongation, toughness, and ultimate strength as CF content increased. This was attributed to defects produced in the polymer matrix by filler dislocation during mechanical testing. The Young’s Modulus was increased to 1.76 MPa (>50%) with the addition of 4 wt.% CF filler, compared with 1.13 MPa of PDMS pristine. In addition, the both the initial and repeated tension performance showed an apparent maximum/steady state Young’s Modulus, which was reached with the 2 wt.% sample. Furthermore, this sample had a relatively small loss of toughness, making it an optimal sample. This observation provides a good guideline for users to modify the mechanical properties of these materials according to specific applications. With the addition of 2 wt.% of CF filler in PDMS, the CF/PDMS composite showed good dimensional stability with a significant decrease in the swelling ratio. This is attributed to the CF filler acting as a physical crosslinker in the PDMS matrix. Thermal degradation temperatures increased by about 25 °C with increasing CF loading in PDMS. Even at 0.5 wt.% CF filler, a 20% reduction in the CTE was observed. Given the results presented in this study, Sylgard™ 186 filled with carbon nanofibers with between 0 and 4.0 wt.% loadings can be tuned to specific applications. CF loadings between 1.0 and 2.0 wt.% in the PDMS polymer matrix present good advantages for applications that require fabrication processes with fine control of their rheological properties, and CF loadings between 2.0 to 4.0 wt.% would work well in areas that require good mechanical properties (e.g., hardness and Young’s Modulus), dimensional stability, and higher thermal degradation. Therefore, the present work provides a good guideline for users to tune Sylgard™ 186 with carbon nanofibers.

## Figures and Tables

**Table 1:** Tension data for PDMS pristine and CF-filled PDMS.

	Elongation (%)	Ultimate Strength (MPa)	Toughness (MPa)	Young’s Modulus (MPa)	
				Initial	After 100% Elongation
PDMS pristine	461	4.92	8.44	1.13	1.13
CF-0.5/PDMS	394	3.95	6.41	1.48	1.42
CF-1.0/PDMS	422	4.48	8.03	1.65	1.52
CF-2.0/PDMS	356	3.60	5.88	1.72	1.53
CF-4.0/PDMS	335	3.47	5.87	1.76	1.52

**Table 2:** Thermal degradation behaviors of PDMS pristine and CF/PDMS samples.

	T_d_ (5% Wt. Loss) (°C)	Degradation Peak 1 (°C)	Degradation Peak 2 (°C)	Degradation Peak 3 (°C)
PDMS pristine	410.9	298.6	491.0	620.4
CF-0.5/PDMS	415.7	317.5	479.4	625.7
CF-1.0/PDMS	420.4	309.7	478.2	624.0
CF-2.0/PDMS	430.4	315.9	484.7	623.1
CF-4.0/PDMS	437.8	314.2	490.1	622.3

**Table 3:** Physical Properties of PDMS pristine and CF/PDMS samples by DSC.

	T_g_ (°C)	T_m_ (°C)	ΔH_f_ (J/g)	T_c_ (°C)	ΔH_c_ (J/g)	Χ_c_ (%) *
PDMS pristine	−123.3	−47.5	23.7	−78.4	24.1	38.7
CF-0.5/PDMS	−123.6	−47.7	23.8	−80.7	23.2	38.9
CF-1.0/PDMS	−123.2	−47.8	23.6	−78.9	23.1	38.6
CF-2.0/PDMS	−121.0	−46.8	23.3	−77.4	25.0	38.1
CF-4.0/PDMS	−123.3	−46.05	23.4	−77.4	23.1	38.2

* Calculated using 61.19 as the enthalpy of fusion for a fully crystalline PDMS polymer [35].

## Data Availability

The data used to support the findings of this study are available from the corresponding author upon request.
